# The clinical, radiological, and surgical characteristics of anterior perforated substance glioma: a retrospective study

**DOI:** 10.1186/s41016-023-00349-w

**Published:** 2023-12-19

**Authors:** Zhiliang Wang, Lianwang Li, Zheng Wang, Xuzhu Chen, Zhong Zhang

**Affiliations:** 1 Department of Neurosurgery, Beijing Tiantan Hospital, Capital Medical University, No. 119 South 4th Ring West Road, Beijing, 100070 People’s Republic of China; 2 Department of Molecular Neuropathology, Beijing Neurosurgical Institute, Capital Medical University, Beijing, China; 3 Department of Radiology, Beijing Neurosurgical Institute, Capital Medical University, Beijing, China; 4 Department of Radiology, Beijing Tiantan Hospital, Capital Medical University, No. 119 South 4th Ring West Road, Beijing, 100070 People’s Republic of China

**Keywords:** Anterior perforated substance, Glioma, Magnetic resonance imaging, Operation

## Abstract

**Background:**

To explore the clinical, radiological, and surgical characteristics of anterior perforated substance (APS) gliomas.

**Methods:**

Twenty patients with APS glioma who were treated with surgery between March 2019 and January 2022 from Tiantan hospital were retrospectively reviewed. The clinical, histological and radiological data were collected.

**Results:**

Twenty patients, including 7 males (55%) and 13 females (45%), with a mean age at diagnosis of 37.9 years (range, 28–53 years) underwent operative intervention for APS. Headaches and dizziness were the most common preoperative symptoms in the majority patients (14, 70%). Based on radiological features of MRI, the APS was classified into two subtypes, type A and type B. Seven patients (40%) in type A indicated a clear tumor margin, while 13 patients (60%) in type B showed an ill-defined margin. The surgical approach including frontal, temporal, and coronal frontal incisions for type A and type B tumors, respectively. Three patients in type A received total resection, while one patient in type B were total resected. Pathologically, 12 cases (60%, 12/20) were diagnosed as astrocytoma and 8 cases (20%, 8/20) were oligodendroglioma. Meanwhile, 17 cases (85%, 17/20) had MGMT promotor methylation.

**Conclusion:**

In this study, we performed the first systematic research of patients with APS glioma. Most of patients with APS presented headaches and dizziness symptoms. The APS glioma was further divided into two major radiological subtypes with relevant different surgical approaches. The APS glioma in type A were more likely to receive total resection.

## Background

The anterior perforated substance (APS) is a special anatomical area. It is a small area with only 23.3 ± 3.4 mm (19–27) in the mediolateral and 12.5 ± 1.2 mm (11–14) in the anteroposterior directions [1]. Although the size is inconspicuous, it contains many important structures, such as anterior perforating arteries and lenticulostriate arteries [2]. These arteries are essential sources of blood supply for some important structures, such as the internal capsule, putamen, and caudate nucleus [3].

Glioma is the most common malignant brain tumor in the central nervous system [4]. The anatomic location of glioma influenced the progression and outcome of patients [5]. However, gliomas in APS have not been well characterized in recent research. Therefore, we conducted this study and aimed to explore the characteristics of patients with APS gliomas.

## Methods

This study was a retrospective study. The study was approved by the ethics committee of Beijing Tiantan Hospital. The clinical information of 20 patients with APS glioma were collected between March 2019 and January 2022. All patients underwent surgery and were pathologically confirmed. As shown in Table 1, among the 20 patients, 11 patients were male and nine patients were female. The mean age was 37.90 ± 7.00 years old, ranging from 28 to 53 years old.

**Table 1:** The Clinical information of APS patients

No.	Age/Sex	Symptom	Surgical approach	Extent of resection	Pathology	MGMT methylation	IDH mutation	1p/19q deletion	TERT mutation
1	28/Female	Epilepsy	Right frontal coronal incision	Subtotal resection	Astrocytoma (WHO II)	Yes	Yes	No	No
2	29/Male	Headache	Right frontal coronal incision	Subtotal resection	Astrocytoma (WHO III)	Yes	Yes	No	No
3	30/Male	Olfactory hallucination	Right frontal and temporal incision	Total resection	Astrocytoma (WHO II)	Yes	Yes	No	No
4	30/Female	Epilepsy	Right frontal coronal incision	Subtotal resection	Oligodendroglioma (WHO III)	Yes	Yes	Yes	Yes
5	33/Male	Blurred vision of the right eye	Anterior interhemispheric approach	Near total resection	Astrocytoma (WHO II)	Yes	Yes	Yes	No
6	34/Male	Personality changes	Right frontal and temporal incision	Subtotal resection	Astrocytoma (WHO II)	Yes	No	No	No
7	34/Male	Epilepsy	Left frontal coronal incision	Subtotal resection	Astrocytoma (WHO II)	Yes	Yes	No	No
8	34/Male	Epilepsy	Left frontal coronal incision	Subtotal resection	Anaplastic Oligodendroglioma (WHO III)	Yes	Yes	Yes	Yes
9	35/Male	Headache	Left frontal and temporal incision	Total resection	Astrocytoma (WHO II)	No	Yes	No	No
10	36/Female	Absence seizure	Left frontal and temporal incision	Subtotal resection	Oligodendroglioma (WHO III)	No	No	No	No
11	36/Female	Headache and dizzy	Left frontal coronal incision	Subtotal resection	Astrocytoma (WHO II)	Yes	Yes	No	No
12	40/Female	Headache	Left frontal coronal incision	Subtotal resection	Oligodendroglioma (WHO III)	Yes	Yes	Yes	Yes
13	40/Female	Dizzy	Left frontal and temporal incision	Subtotal resection	Astrocytoma (WHO II)	Unavailable	Yes	Unavailable	Unavailable
14	41/Female	Headache	Left frontal coronal incision	Subtotal resection	Astrocytoma (WHO II)	Yes	Yes	No	Unavailable
15	42/Male	Epilepsy	Left frontal coronal incision	Subtotal resection	Astrocytoma (WHO II)	Yes	Yes	No	Unavailable
16	43/Male	Epilepsy	Left frontal coronal incision	Subtotal resection	Oligodendroglioma (WHO III)	Yes	Yes	Yes	Yes
17	43/Male	Headache	Right frontal and temporal incision	Total resection	Astrocytoma (WHO II)	Unavailable	Yes	Unavailable	Unavailable
18	47/Female	dizzy	Left frontal coronal incision	Subtotal resection	Astrocytoma (WHO II)	Yes	Yes	No	Unavailable
19	50/Female	Headache	Left frontal coronal incision	Subtotal resection	Oligodendroglioma (WHO III)	Yes	Yes	Yes	Yes
20	53/Female	Headache and dizzy	Left frontal coronal incision	Subtotal resection	Astrocytoma (WHO II)	Yes	No	No	No

All patients underwent magnetic resonance imaging (MRI) scanning before surgery. The MRI sequence included pre-contrast T1-weighted image(T1WI), T2-weighted image(T2WI), fluid attenuated inversion recovery (FLAIR), diffusion weighted image (DWI), and post-contrast T1WI. The MRI scanners used were SIEMENS Verio (5 cases), SIEMENS Prisma (3 cases), Philips Ingenia CX (2 cases), Philips Ingenia (4 cases), GE SIGNA Explorer (2 cases), and GE DISCOVERY MR750 (4 cases). The thickness and gap of scanning were 5.00–5.50 mm and 6.00–6.50 mm, respectively.

**Fig. 1:**
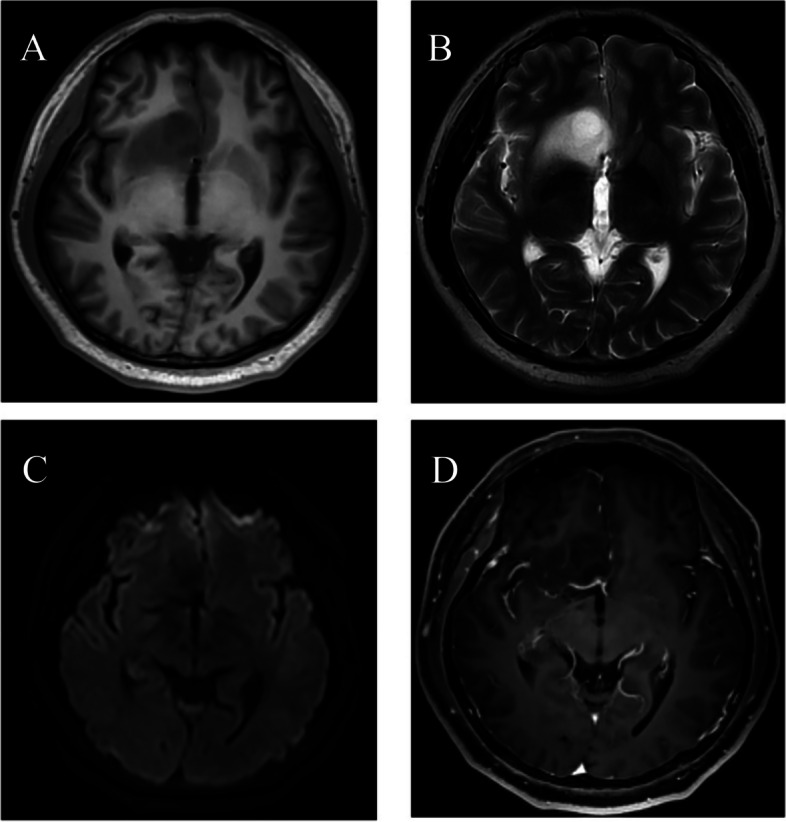
33-year-old male patient with type A anterior perforated substance (APS) glioma. The lesion involves the right posterior part of straight gyrus, the anterior and inferior part of basal ganglia, and the medial of insula. It is low signal on T1-weighted image (T1WI) (**A**), high signal on T2-weighted image (T2WI) (**B**), low signal on diffusion weighted image (DWI) (**C**), and no enhancement (**D**)

**Fig. 2:**
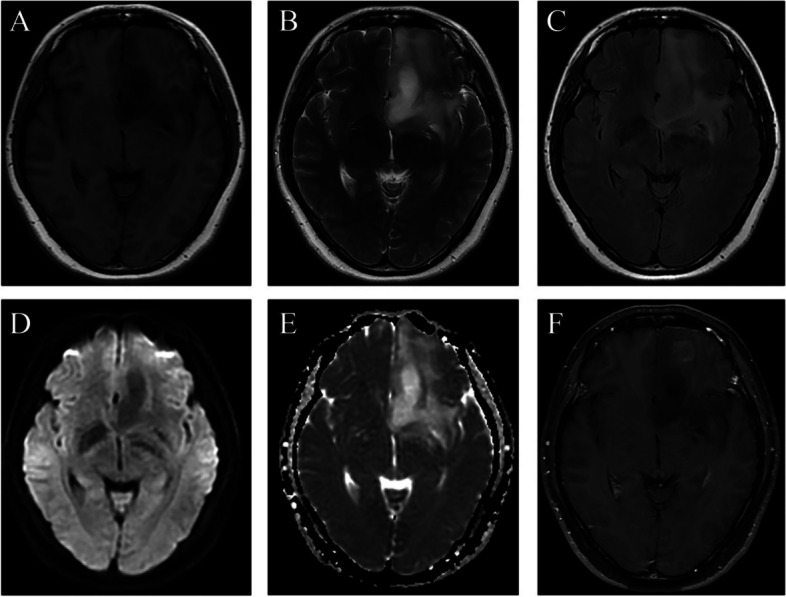
36-year-old female patient with type B APS glioma. The lesion involves the left temporal pole, orbital gyrus, straight gyrus, paraolfactory gyrus, and paraterminal gyrus. It is low signal on T1WI (**A**), high signal on T2WI (**B**) and fluid attenuated inversion recovery (FLAIR) (**C**), low signal on DWI (**D**) and high signal on apparent diffusion coefficient (ADC) (**E**). On post-contrast axial image, it demonstrates patchy enhancement (**F**)

According to the preoperative MRI, the APS gliomas were divided into two types: type A, the tumor mainly located in the APS, showing an expansive growth pattern, while the margin was relatively clear (Fig. 1A-D); and type B, the tumor extended out of the APS, showing a diffuse growth pattern with an ill-defined margin (Fig. 2A-F).

## Results

The clinical characteristics of the 20 patients with APS gliomas were presented in Table 1. The most common symptom was headache with or without dizziness (70%, 14/20), followed by epilepsy (30%, 6/20). Radiologically, 7 cases (35%, 7/20) were type A and 13 cases (65%, 13/20) were type B.

As shown in Table 2, total resection was performed in 3 patients (42.8%) in type A compared with 1 patient (7.6%) in type B (*p* = 0.101). There was a trend that APS glioma in type A was more likely to obtain total resection. Meanwhile, the molecular analysis indicated that all APS gliomas were IDH mutation and the proportion of 1p/19q codeletion was similar in two groups (*p* = 0.999). Besides, patients in type B were associated with MGMT promoter methylation (92.3%) and TERT promotor mutation (38.4%). In addition, all patients in type A were diagnosed as grade 2 glioma (3 astrocytoma. 3 oligodendroglioma), and nearly half of type B patients were grade 3 glioma (*p* = 0.031). The results indicated that type B APS gliomas were more aggressive and heterogeneous.

**Table 2:** The clinical and histological information of patients

Variable	APS type A	APS type B	*P*.value
Age, years (mean)	38.2	37.6	0.862
Sex			
Male	4	7	0.999
Femal	3	6	
Extent of resection			
Subtotal resection	4	12	0.101
Total resection	3	1	
Pathology			
Astrocytoma (CNS WHO 2)	4	7	0.031
Astrocytoma (CNS WHO 3)	0	1	
Oligodendrocytoma (CNS WHO 2)	3	0	
Oligodendrocytoma (CNS WHO 3)	0	5	
IDH status			
Mutation	7	13	
Wild type	0	0	
1p/19q status			
Codel	3	5	0.999
Intact	4	8	
MGMT			
Methylated	5	12	0.272
Unmethylated	2	1	
TERT promotor status			
Mutation	0	5	0.113
Wild type	7	8	

**Fig. 3:**
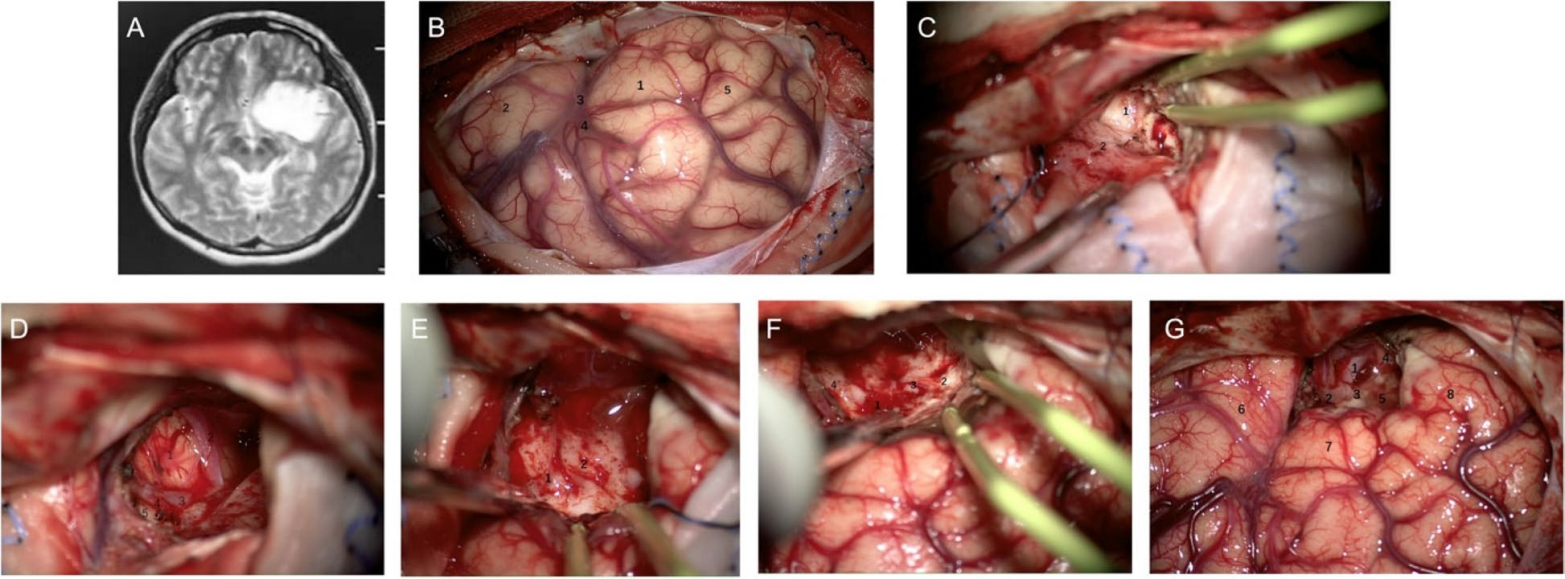
The intraoperative of type A APS glioma. **A**, Preoperative axial T2WI showing type A APS glioma. **B**, Intraoperative view before resection (1. the orbital part of the inferior frontal gyrus (IFG), 2. superior temporal gyrus (STG), 3. sylvian fissure (SyF), 4. anterior sylvian point (ASyP), 5. the middle frontal gyrus). **C**, The lateral margin of resection (1. Olfactory tract and olfactory trigone, 2. Tumor). **D**, Intraoperative view of lenticulostriate artery (1. the planum polare, 2. the early branch of middle cerebral artery (MCA), 3. the M1 segment of the MCA, 4. extraparenchymatous segments of the most lateral lenticulostriate arteries (LSAs), 5. the limen insular). **E**, The medial and lateral margins of the resection (1. intraparenchymatous segment of the most lateral LSAs, 2. tumor in APS). **F**, The posterior and superior margins and the lateral margin of the resection (1. the putamen, 2. the head of the caudate nucleus, 3. The lenticulostriate veins, 4. the limen insular). **G**, Intraoperative view after resection (1. Internal carotid artery, 2. M1, 3. A1, 4.CN II, 5. APS, 6. superior temporal gyrus (STG), 7. the posterior part of the inferior frontal gyrus (IFG), 8. the middle frontal gyrus)

In order to achieve maximum safe resection, different surgical approaches were performed. For type A glioma, surgical approaches of frontal and temporal incisions were used (Fig. 3A). After resection of the lateral orbital and the partial inferior frontal gyri, the gray tumor, olfactory nerve, and olfactory trigone could be exposed. The olfactory trigone was considered the frontal margin of surgical resection (Fig. 3B). The tumor was then removed from the lateral olfactory stria to the insula with exposure of the internal carotid artery, M1 segment of the middle carotid artery, and insula. When the outside lenticulostriate artery originating from M1 penetrating the APS was confirmed, the lateral margin of resection could be determined (Fig. 3C). The medial margin of resection may be the anterior median fissure, and the genu of the corpus callosum and A2 segment of the anterior carotid artery may be the anatomical signs. When resecting the lateral part of the tumor, the operation should be as softly as possible. We recommended to remove the tumor with aspirator one by one layer (as thick as 1 mm) and avoid using the electrocoagulation. As shown in the Fig. 3D, we can see the lenticulostriate artery penetrated the tumor during the surgery. When the posterior and superior parts of the tumor were resected, the amplification and brightness of the microscope should be adjusted. Through applying intraoperative neuro-navigation, the caudate nucleus and putamen could be identified, which showed bright and cream-colored spots under microscope (Fig. 3E). When these structures were exposed, the medial and lateral margins of the resection could be confirmed. The caudate nucleus (white spots on a gray/red background) and putamen (white stripes and dots of beige color) were the posterior and superior margins and the lateral margin of the resection (Fig. 3F). As shown in Fig. 3G, the anatomical structures of the APS could be clearly identified after the tumor resection.

For tumors of type B, glioma invasion of frontal lobe, the surgical approach was coronal frontal incisions. The medial margin of the craniotomy was approximately 1–1.5 cm lateral to the midline. The posterior margin of the craniotomy was anterior to the coronal suture. The lateral margin of the craniotomy reached sphenoid ridge to expose the sylvian fissure. The anterior margin of the craniotomy could be performed just at the level of the skin flap exposure and be adjusted according to the size of the frontal sinus. We avoided opening the frontal sinus as much as possible.

## Discussion

APS is a special anatomical area, which contains many vessels [6]. Previous studies involving APS mainly focused on vascular diseases [3, 7–9]. Glioma, one of the most common malignant tumors in the central nervous system, can also locate in APS. However, gliomas in this area have not been well described. Our retrospective study revealed that APS gliomas differed from those in other areas in terms of clinical, radiological, and surgical characteristics.

In our study, patients with APS gliomas showed a slightly male predominance, which was in consistent with the overall population characteristics of patients with gliomas reported by Li et al. [10]. The mean age of patients with APS gliomas was 37.90 ± 7.00 years old, which was younger than 60 years old [11]. This discrepancy may be caused by the small sample size of our study; Moreover, the low tumor grade of APS gliomas in our study may play a part, as the mean age increased with the growing of glioma grade [11]. Regarding to symptoms, the most common symptom in patients with APS gliomas were headache with or without dizziness (70%, 14/20), which were different from those reported by Rasmussen et al. and IJzerman-Korevaar et al. [11, 12]. This may be a unique clinical characteristic of APS gliomas. Due to the small sample size of our study, the reliability needs to be further verified in the future.

Radiologically, APS gliomas show hypointensity on T1WI, hyperintensity on T2WI and T2 FLAIR, nonrestricted diffusion and partial enhancement on post-contrast T1WI. These radiological characteristics were not specific for gliomas. It’s worth pointing out that APS gliomas can be divided into two subtypes according to their morphological appearances on radiological images (type A, localized tumor with well-defined margins; type B, diffused tumor with ill-defined margins). This artificial classification is helpful for tumor resection and cognition of the relationship between gliomas and adjacent structures in APS.

The surgical resection of glioma in APS is a challenge for neurosurgeons due to the special anatomic characteristics. On the one hand, the determination of the depth and extent of resection is difficult. Tumor exposure and determination of the extent of resection are big challenges for APS glioma. APS is overlapped by the uncinate gyrus, olfactory trigone, optic nerve, and optic tract. Thus, it is difficult to fully expose APS. And anatomical landmarks were needed to determine the extent and depth of resection. For example, the olfactory trigone is an important landmark for determining the anterior margin of APS. Restricted by the drift of brain tissue, intraoperative navigation is not completely reliable. Therefore, gray matter identification is of great importance during surgery; On the other hand, the exposure and protection of penetrating arteries are challenging, especially the lateral lenticulostriate arteries.

Our study showed that the pathological diagnosis of all patients with APS gliomas were lower grade gliomas Furthermore, all APS gliomas possessed IDH mutations, which was higher than the general ratio of 45% in gliomas reported by Eckel-Passow et al. [13, 14]. The ratio of MGMT methylation was 85% (17/20), which means the increased sensitivity to chemotherapy and longer patient survival [14, 15]. The ratio of TERT mutations in our study was 25% (5/20), which was higher than that obtained in the study by Killela et al. [16]. The genetic status of APS gliomas provides positive information for patients because IDH mutation in gliomas has been a treatment target [17–19]. Patients with IDH-mutated gliomas show better prognosis [18–20], while patients with TERT-mutated glioma show compromised overall survival and progression-free survival [21].

Our study had some limitations. First, as a retrospective study, selective bias may exist, and the sample size was not large. Further study with more cases is needed in the future. Second, the APS patients were IDH mutation lower grade glioma with the longest follow-up nearly 4 years. Detailed information of patients with APS gliomas after surgery should be supplemented in the future. Third, except for routine MRI and DWI, more advanced imaging modalities should be performed for the tumors.

In conclusion, our study showed that gliomas in APS have different clinical, radiological, and pathological characteristics. Tumors can be divided into two types according to the radiological patterns with relevant different surgical approaches. Moreover, most APS gliomas are low-grade gliomas with a high ratio of IDH and TERT mutations and MGMT methylation. Due to the special anatomical characteristics of APS, more research is needed on gliomas in this area.

## Data Availability

The sequencing data, clinical, and follow-up information of patients were uploaded to the CGGA portal (http://cgga.org.cn/). All datasets used and/or analyzed in this study are available from the corresponding author on reasonable request.

## Abbreviations

APS: Anterior perforated substance
DWI: Diffusion weighted image
FLAIR: Fluid attenuated inversion recovery
MRI: Magnetic resonance imaging
T1WI: T1-weighted image
T2WI: T2-weighted image
